# Wheel-Rail Contact-Induced Impact Vibration Analysis for Switch Rails Based on the VMD-SS Method

**DOI:** 10.3390/s22186872

**Published:** 2022-09-11

**Authors:** Pan Hu, Haitao Wang, Chunlin Zhang, Liang Hua, Guiyun Tian

**Affiliations:** 1 School of Electrical Engineering, Nantong University, Nantong 226019, China; 2 College of Automation Engineering, Nanjing University of Aeronautics and Astronautics, Nanjing 211106, China; 3 COSCO SHIPPING shipyard (Nantong) Co., Ltd., Nantong 226019, China

**Keywords:** impact vibration analysis, VMD, SS, velocity effect, wheel—rail contact

## Abstract

When trains pass through damaged switch rails, rail head damage will change wheel–rail contact states from rolling frictions to unsteady contacts, which will result in impact vibrations and threaten structural safeties. In addition, under approaching and moving away rolling contact excitations and complex wheel–rail contacts, the non-stationary vibrations make it difficult to extract and analyze impact vibrations. In view of the above problems, this paper proposes a variational-mode-decomposition (VMD)-spectral-subtraction (SS)-based impact vibration extraction method. Firstly, the time domain feature analysis method is applied to calculate the time moments that the wheels pass joints, and to correct vehicle velocities. This can help estimate and confine impact vibration distribution ranges. Then, the stationary intrinsic mode function (IMF) components of the impact vibration are decomposed and analyzed with the VMD method. Finally, impact vibrations are further filtered with the SS method. For rail head damage with different dimensions, under different velocity experiments, the frequency and amplitude features of the impact vibrations are analyzed. Experimental results show that, in low-velocity scenarios, the proposed VMD–SS–based method can extract impact vibrations, the frequency features are mainly concentrated in 3500–5000 Hz, and the frequency and peak-to-peak features increase with the increase in excitation velocities.

## 1. Introduction

Switch rails enable trains to change tracks, which affect the smooth and safe operation of train systems and therefore play an important role in railway infrastructures [1,2]. The braking and starting behaviors of trains when passing switch rails and complex wheel–rail contacts will lead to large contact stress. When the contact stress exceeds the switch rail yield strength, fatigue cracks or spalling will occur at rail heads [3]. The existence of damage will affect wheel–rail contact states from rolling friction to unsteady contact, and corresponding impact vibrations will occur [4]. The features of impact vibration can be several times those of conventional rolling friction, and are accompanied by large impact accelerations [5]. These pose serious threats to the structural integrities of rails and wheelsets. Therefore, monitoring and analyzing impact vibrations is of great significance to ensure the safe operation of trains in switch rail areas.

At present, there are mainly three types of techniques for rail vibration analysis: time-domain-, frequency-domain- and time–frequency-domain-based techniques. (1) Time domain analysis technology is mainly divided into two types: the structural modal feature extraction and the damage feature extraction. Among them, the structural modal feature extraction technology mainly includes an eigensystem realization algorithm, a natural excitation technique and an auto-regressive and moving average model [6,7,8]. Based on the above methods, references [6,7,8] realized dynamic parameter extractions and analysis for damaged rails. However, the eigensystem realization algorithm has low interference immunities, as the natural excitation technique needs large calculation amounts. In addition, when system orders cannot be determined, they may identify false modes. The auto-regressive and moving average model requires vibration signals to be stable, which cannot be met in practical conditions. Meanwhile, the above methods are not sensitive to local damage. Therefore, some researchers propose to use damage feature extraction technology to detect damage [9,10], e.g., the peak-to-peak value, kurtosis and margin. These features are derived from the original vibrations and contain complete structural state information. However, the time-domain feature extraction technique requires a high signal-to-noise ratio of vibrations. This also cannot be met in practical conditions. (2) Frequency domain analysis technology mainly includes [9] a Fourier transform and frequency spectrum analysis, which have been proved to be effective in rail geometric irregularity [11] and rail fastener state analysis [12] applications. However, the signal used for frequency domain analysis also needs to have high signal-to-noise ratios. Meanwhile, this method is used to analyze the entire vibration data segment and cannot analyze the local frequency component changes caused by damage. (3) The time–frequency domain analysis technology mainly includes [13,14] the wavelet transform and Hilbert–Huang transform. The influence of wheelset creep [15] and wheel flatness [16] based on rail vibrations has been successfully studied. However, the wavelet transform is more suitable for linear problems, and the Hilbert–Huang transform is prone to modal aliasing [17].

In sum, the above three type technologies have challenges for non-stationary signal processing and are insensitive to local damage [18,19], which will affect the accuracy of the extraction and analysis for impact vibrations. To solve these problems, researchers have proposed a signal stabilization processing method: Li [20] applied a combination of empirical mode decomposition (EMD) and the state probability method to predict track irregularity states with different wavelengths; Wang [21] integrated ensemble empirical mode decomposition (EEMD) and constrained independent component analysis to realize rail-damage-induced signal extractions; Du [22] proposed a detecting mechanism of local mean decomposition (LMD) energy, a moment-directed acyclic graph support vector machine for train-wheelset-bearing diagnoses. However, when using EMD, EEMD and LMD for non-stationary impact vibration processing, there still exist problems of modal aliasing, the end effect and the inability to separate components with similar center frequencies [23].

VMD is a new signal stabilization processing and decomposition method. Compared with EMD, EEMD and LMD, VMD converts signal decomposition into non-recursive and modal variation, which shows better robustness. In terms of modal decomposition, VMD can identify signal components with similar frequencies. Therefore, VMD has received extensive attention in rail vibration processing applications. Zhang [24] takes an envelope entropy of decomposed vibration signals by VMD as an index to detect wavelengths and positions of rail corrugation damage. Chen [25] studies the influence of train velocity on rail high frequency vibrations through VMD and a marginal spectrum. However, there is no research on applying VMD to extract and to further analyze impact vibrations for switch rails. In addition, the stabilization-processed signals may still contain stationary noises, which will cause defect-related signals to be masked. Iglesias-Martínez [26] and Balaji [27] have verified that SS is helpful in noise reductions and further signal extractions. Considering the above, the SS method can be a candidate method to further extract impact vibrations.

In this paper, a VMD-SS signal processing method is proposed to extract and analyze damage-induced impact vibrations. Firstly, the moment when the wheelset passes rail joints is determined through the time domain analysis method. This helps determine the distribution range of the impact vibrations in the time domain. Secondly, a stable IMF component containing impact vibration is obtained through VMD. Then, the impact vibration component is extracted through filtering stable noise with SS. Based on the wavelet time–frequency analysis method, the effects of velocities and damage dimensions on impact vibration features are analyzed under different velocity experiments.

The remainder of this paper is organized as follows: Section 2 gives a detailed introduction to the proposed method. Section 3 describes the experimental study results. After a discussion in Section 4, the conclusion and future works are considered in Section 5.

## 2. Extracting and Analyzing Methods for Impact Vibration Signals

**Figure 1:**
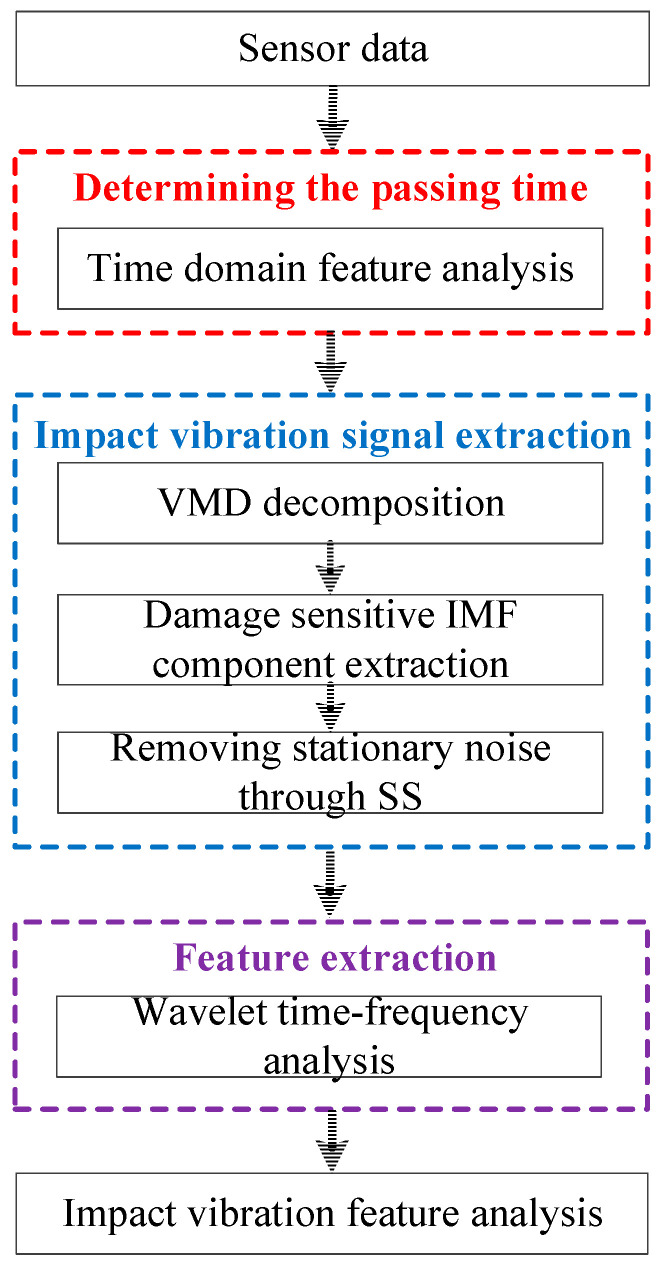
The flow chart of signal processing.

This section describes the signal processing procedure of the proposed method. Figure 1 shows the overall diagram; it mainly includes three steps: (1) determining the passing time: extracting and analyzing the peak-to-peak features of the raw vibrations in the time domain and determining the time moment when the vehicle passes the joint; (2) impact vibration extraction: optimizing the VMD decomposition layer number through the center frequency selection and then removing stationary noise through SS to extract impact vibrations; (3) extracting features: extracting impact vibration features through the wavelet time–frequency analysis method. Among the three steps, step 1 will be introduced in Section 3.1 and the details concerning the processing and calculating for step 2–3 will be introduced in Section 2.1 and Section 2.2.

### 2.1. Impact Vibration Signal Extraction Method

This step mainly includes VMD and SS. The processing flow of VMD is as follows: (1) setting the initial decomposition layer number K, the penalty factor α and bandwidth parameter τ to the default parameters of 2, 200 and 0, respectively; (2) calculating center frequencies for each IMF layer, and comparing whether each of the two layer center frequencies are too close or too far. If it does not meet the above requirements, switch to step (3); otherwise switch to step (4); (3) K=K+1, switch to step (2); (4) selecting an impact-sensitive IMF component through signal analysis. The detailed principles and more introductions of VMD are [28]:

VMD searches for the optimal solution of the variational model iteratively, so as to determine the center frequency and bandwidth of each decomposed IMF component. This can realize adaptive sparse signal decompositions. The bandwidth of the decomposed mode’s center frequency is constantly updated to seek a minimum mode function $u_{k}\left(t\right)$ during the decomposition process, so that the sum of each IMF component is equal to the input signal. The main sub steps [26] are: (1) obtaining the unilateral spectrum of each component; (2) adjusting the frequency spectrum of the IMF component to baseband through introducing an exponential function; (3) establishing the optimal variational model. Then, the optimal solution is obtained through the variational model, and the raw input signal is decomposed into finite IMF components.

After decomposing and extracting the IMF components from the impact vibration, the next step is to use the SS method [29] to subtract the noise power spectrum from the extracted IMF component power spectrum to obtain the impact vibrations. Suppose y(n) is an IMF component that contains both noise and impact vibration, s(n) is the impact vibration part and d(n) is the noise part. Then, the relationship among the three parts can be expressed as:

(1) y(n)=s(n)+d(n), 0≤n≤N−1

where n is the number of data points and N is the frame length.

Taking the Fourier transform for both sides of Equation (1) and calculating the corresponding short time power spectra, they are named Y(ω), S(ω) and D(ω), respectively. Then, for the short-term stationary process of an IMF component:

(2) $${\left|S\left(\omega \right)\right|}^{2}={\left|Y\left(\omega \right)\right|}^{2}-\lambda_{d}\left(\omega \right)$$

where $\lambda_{d}\left(\omega \right)$ is the statistical mean of ${\left|D\left(\omega \right)\right|}^{2}$ in silent sections. Here, we applied the common method of setting the first five frames as an interval of silence and set the average of the spectra to that of the noise spectrum [30].

Therefore, the final extracted impact vibration can be expressed as:

(3) $$\begin{array}{ll} \hat{S}\left(\omega \right) & ={\left[{\left|Y\left(\omega \right)\right|}^{2}-E\left({\left|Y\left(\omega \right)\right|}^{2}\right)\right]}^{1/2}\\ & ={\left[{\left|Y\left(\omega \right)\right|}^{2}-\lambda_{d}\left(\omega \right)\right]}^{1/2} \end{array}$$

### 2.2. Feature Analysis of Impact Vibration

After extracting impact vibration through VMD and SS, the next step is to extract and analyze the features with the wavelet transform. The discrete wavelet transform has the advantages of small calculations and a fast analysis. Therefore, it is suitable and is used to extract the frequency feature of impact vibrations in this section. The main process of wavelet transform can be expressed as [31]:

The continuous wavelet transform of a specified signal f(t) can be defined as:

(4) $$W_{\psi }^{f}\left(a,b\right)=\frac{1}{\sqrt{a}}\int_{-\infty }^{+\infty }f\left(t\right)\psi^{*}\left(t\right)dt$$

where $\psi_{a,b}\left(t\right)=\psi \left(\left(t-b\right)/a\right)/\sqrt{a}$, $\psi^{*}\left(t\right)$ is the mother wavelet function, a is the scale parameter and b is the translation parameter. For the parameters of the discrete set, $a=2^{-j}$, $b=2^{-j}k$, j,k∈Z, the wavelet basis is:

(5) $$\psi_{j,k}\left(t\right)=2^{j/2}\psi \left(2^{j}t-k\right)$$

Therefore, the discrete wavelet transform of a specified signal f(t) can be defined as:

(6) $$wt\left(j,k\right)=\langle f\left(t\right),\psi_{j,k}\left(t\right)\rangle =2^{j/2}\int_{-\infty }^{+\infty }f\left(t\right)\psi^{*}\left(2^{j}t-k\right)dt$$

where 〈·〉 is the inner product, expressed as $\langle f\left(t\right),\psi_{j,k}\left(t\right)\rangle =\int f\left(t\right)\psi_{j,k}^{*}\left(t\right)dt$.

Before verifying the proposed signal processing method, it is necessary to analyze the energy distribution principle of the impact vibration to optimize the sensor array arrangement. This can improve the detectability of impact vibration signals.

### 2.3. Optimizing Sensor Arrangements

**Figure 2:**
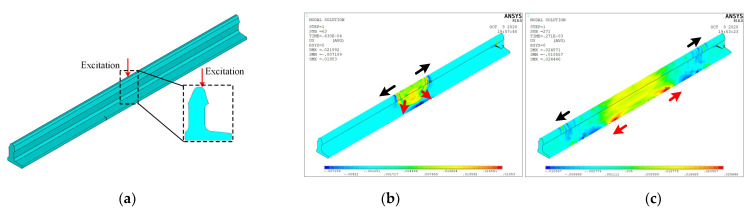
Finite element simulation model and results: (**a**–**c**).

In this section, the finite element analysis software ANSYS 18.0 (Ansys, Inc., Nantong, China)is used to optimize the sensor array arrangement from the perspective of simulating the vibration energy distribution principle under impact excitations at a switch rail head. As shown in Figure 2, a three-dimensional finite element switch rail model is established through the three-dimensional solid structural unit SOLID187. The switch rail model is CHN60AT, and the model length is two meters. The corresponding material parameters are shown in Table 1. To help observe the energy distribution principle and to reduce reflections, the excitation is selected at the center part of the switch rail head, which is shown as the red arrow in Figure 2a. Zhai [5] determined that the frequency band of the impact vibration can be several times the normal load induced vibration, and the instant contact state between the damage and wheelsets is similar to an impact excitation source. In addition, Zhai [32,33] also investigated the dynamic performance of rails under track irregularity scenarios, and the results showed that the vibration frequencies are mainly concentrated in less than 200 Hz. This ensures that the track irregularity will not influence the impact vibrations. Therefore, in this section, a half-cycle sinusoidal signal with a center frequency of 3 kHz is selected to simulate an impact excitation:

(7) $$f\left(t\right)=\left\{ \begin{array}{cc} 0.1\sin\left(\frac{2\pi }{T\cdot t}\right)\; & 0\leq t\leq \frac{T}{2}\\ 0 & t>\frac{T}{2} \end{array}\right.$$

where *T* is the period of the 3 kHz sine function.

The simulation is completed on the workstation with the main parameters of Inter (R) Core (TM) i9-9900, 64 GB memory and 64-bit Windows 10 Professional operating system. The simulation results are sampled at a time interval of 10 μs to obtain vibration energy distribution cloud maps, and the time length of the simulation is 1 ms. The cloud maps are illustrated in Figure 2b,c. It can be observed that the vibration energies generated by the impact excitation from the switch rail head are distributed at the rail head, web and base. In addition, when the vibration energy propagates to the rail base, it will continue to propagate along the rail base to both sides of the model. Considering that the sensors cannot be arranged at the rail head when the switch rail is in service, arranging the sensor array at the rail base will be helpful for impact vibration acquisition and structural state monitoring.

## 3. Experimental Study and Results

The experimental research in this section was conducted on an in-service switch rail section. A rail inspection vehicle was taken as the excitation, and the passing time, impact vibration extraction and feature analysis were studied, respectively.

### 3.1. Experiment Scheme and System

The experimental study was conducted at Gemac Engineering Machinery Co., Ltd., which is located in Xiangyang City, China. The monitoring object was the head damage of the switch rails in the test rail line. The vehicle was jointly developed by the China Academy of Railway Sciences and Gemac Engineering Machinery Co., Ltd. When the inspection vehicle passes the rail damage, the wheel–rail contact states change to unsteady contacts and corresponding impact vibrations are generated [5]. To ensure safety, it should be noted that the velocities in the factory were limited to less than 20 km/h.

**Figure 3:**
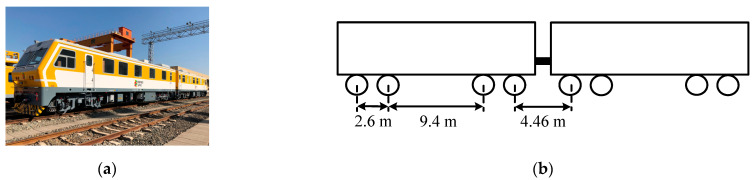
The track inspection car and wheelset distances: (**a**) track inspection car; (**b**) wheelset distance parameters of track inspection.

The figure and parameters of the vehicle are shown in Figure 3a,b. The vehicle included two locomotives, and the distance between the two locomotives was 4.46 m. The weight of each locomotive was 67 t. Each locomotive included two bogies, and the distance between the two pair of wheelsets in a bogie was 2.6 m, and the distance between the front and the back bogies was 9.4 m. The above distance parameters were used to determine the vehicle velocity and to locate the time when the wheelset passed the joint in the subsequent chapters.

**Figure 4:**
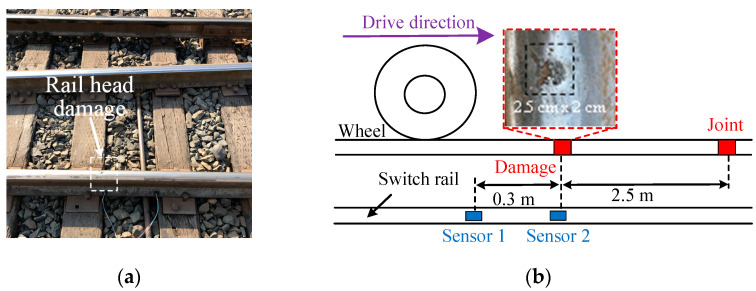
Target damage and experimental scheme: (**a**) target damage; (**b**) experimental scheme.

The experimental scheme of this section is shown in Figure 4a,b. The dimension of the detected damage at the switch rail head was about 2.5 cm × 2 cm. The damage was 2.5 m away from the joint, and there was no other obvious damage between the detected damage and the joint in the wheel–rail contact areas. This helped avoid the misjudgment of the impact vibrations caused by other damage. To ensure repeatability, two PZT sensors, which were named sensor 1 and sensor 2, were installed at a 30 cm interval in the vertical direction of the switch rail base. In the experiments, the vehicle passed through the switch rail damage and the joint with different velocities. The preseted velocities were 12 km/h, 14 km/h, 16 km/h, 18 km/h and 20 km/h, respectively.

**Figure 5:**
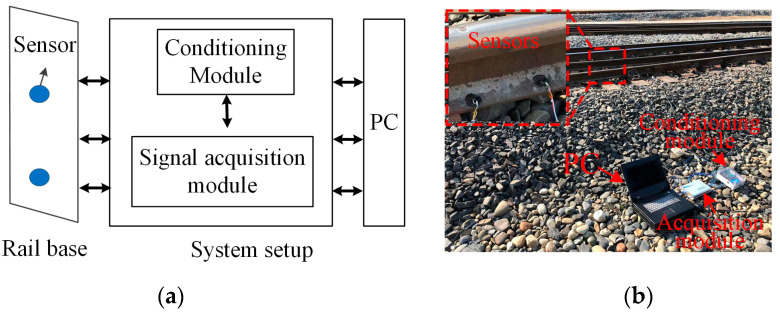
The (**a**) diagram and (**b**) figure of the experimental system.

Figure 5a,b shows the diagram and figure of the experimental system. The system was mainly composed of sensors, a conditioning module, a signal acquisition module and a PC. The professional acquisition device ART USB8514B was selected as the acquisition device in this system, which can realize four-channel synchronous sampling with 40 MSa/s. The sensor model was DS-1201 produced by TE Company; the length, width and height were all 8.9 mm, which made it easy to integrate with the switch rail base. The conditioning module was from the switch rail monitoring system developed by our group [31]. The sampling frequency was set to 10 kHz and the sampling time was 10 s.

### 3.2. Determining Passing Time and Correcting Velocity

**Figure 6:**
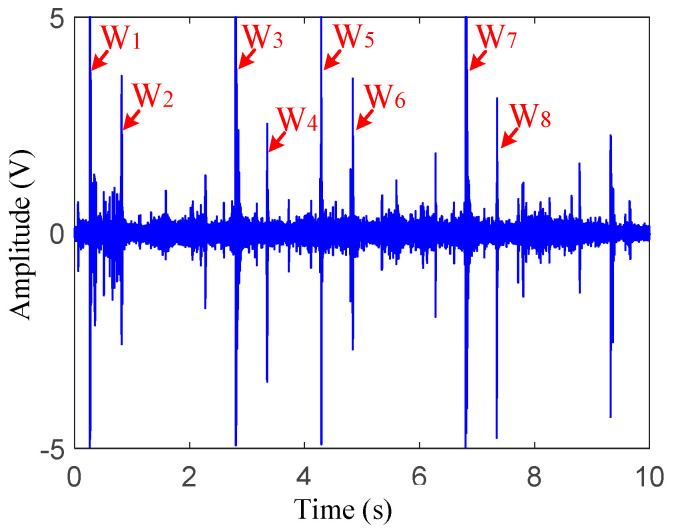
Raw vibration response of measuring point 1 under preseted 16 km/h excitation.

Taking the vibrations of the vehicle passing through the damage and nearby areas at 16 km/h (about 4.44 m/s) as an example, the time domain signals are shown in Figure 6. As shown in this figure, the signal-to-noise ratio of the original signals was low; hence, it was difficult to identify and extract the damage-induced impact vibration directly. Based on Zhai’s research [3] that rail joints can cause strong wheel–rail contacts and that the corresponding vibration index can be several times that of the conventional wheel-rail force, this section takes the point of view of seeking when wheelsets pass through the rail joint, taking this time moment as a benchmark and analyzing the vibrations around this time to assist with the damage-induced impact vibration extractions.

As shown in Figure 6, eight distinct pulses with large amplitudes, which were named *W*_1_–*W*_8_, respectively, could be observed from raw vibration signals. To determine the distances between each of the two adjacent pulses, the appearance time parameters of *W*_1_–*W*_8_ were firstly extracted. Then, based on the preseted 16 km/h velocity, the distances between each two adjacent pulses can be calculated:

(8) $$\left\{ \begin{array}{l} d_{W_{12}}=\left(0.8186\;s-0.2708\;s\right)\times 4.44\;m/s\approx 2.43\;m\\ d_{W_{23}}=\left(2.808\;s-0.8186\;s\right)\times 4.44\;m/s\approx 8.83\;m\\ d_{W_{34}}=\left(3.352\;s-2.808\;s\right)\times 4.44\;m/s\approx 2.4\;m\\ d_{W_{45}}=\left(4.287\;s-3.352\;s\right)\times 4.44\;m/s\approx 4.15\;m\\ d_{W_{56}}=\left(4.839\;s-4.287\;s\right)\times 4.44\;m/s\approx 2.45\;m\\ d_{W_{67}}=\left(6.816\;s-4.839\;s\right)\times 4.44\;m/s\approx 8.78\;m\\ d_{W_{78}}=\left(7.348\;s-6.806\;s\right)\times 4.44\;m/s\approx 2.41\;m \end{array}\right.$$

From the above results, it can be found that the calculated distances under the preseted velocity were approximately equal to the distances between each of the two adjacent wheelsets in Figure 3b. In addition, there were only eight distinct impulses in the time domain signal when the vehicle passed through the monitoring area. Therefore, it can be assumed that the eight pulses in Figure 6 were caused by the wheel–rail impact when the eight wheelsets passed through the joint. To verify this assumption, the corrected velocity was calculated with the time difference between *W*_1_ and *W*_2_: v=2.6 m÷0.5478 s≈4.75 m/s=17.1 km/h. Then, the distances of $d_{W_{23}}$-$d_{W_{78}}$ were recalculated and are illustrated in Table 2. Then, they were compared with the distances in Figure 3b. It can be observed from this table that the calculated distances were basically consistent with the actual distances with small errors, which verifies that the eight pulses were caused by the wheel–rail impacts when the vehicle passed the joint.

### 3.3. Impact Vibration Extraction

In the previous section, the calculated passing time was helpful to determine the impact vibration signal distribution area caused by damage. However, it still can be seen in Figure 6 that the signal-to-noise ratio was low and there were still many other small impulses. These will affect the impact vibration extraction. Therefore, the raw vibration signal needs to be processed by the proposed VMD-SS to help identify and extract the damage-induced impact vibrations. Taking the raw vibration signals of the preseted 16 km/h experiment of sensor 1 as an example, the vibration signal was decomposed through VMD firstly. Before VMD, it was necessary to optimize the IMF component number. Based on the central frequency estimation [28], the default penalty factor, the accuracy and the IMF component numbers were set as 2000, 10 × 10^−6^ and 2–5, respectively. The central frequencies of the different IMF component numbers were calculated and are shown in Table 3. As seen in this table, when the number was two and three, the central frequency difference between the two IMFs was large, which was due to the under decomposition; when the number was five, the center frequencies of IMF3 and IMF4 were close, which was due to the over decomposition. Therefore, the IMF component number was selected as four in this section.

**Figure 7:**
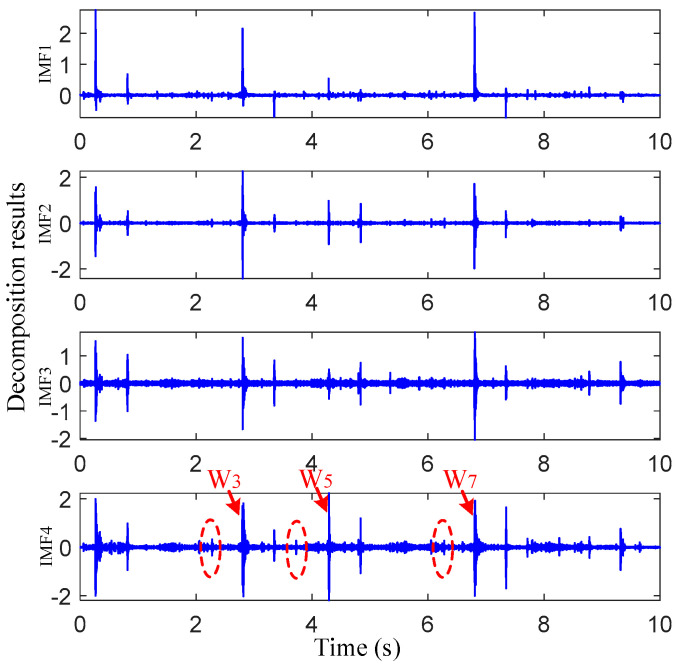
Vibration response of measuring point 1 under 17.1 km/h excitation.

Figure 7 shows the decomposed results for the preseted 16 km/h experiment after VMD. It was observed that several short-time impulses with small amplitudes appeared at the left sides of *W*_3_, *W*_5_ and *W*_7_ in IMF4, which are marked with red dotted lines. The possible physical meaning is that the impact vibrations were generated at a certain distance from the left side of the joint before the third, fifth and seventh wheelsets passed the switch rail area. This is consistent with the fact that the target damage in Figure 4b was located on the left side of the joint. However, the amplitudes of the three candidate impulses were almost equivalent to those of the background noise. To further extract the candidate impact vibrations, the SS method was applied to denoise the IMF4 component in this section.

**Figure 8:**
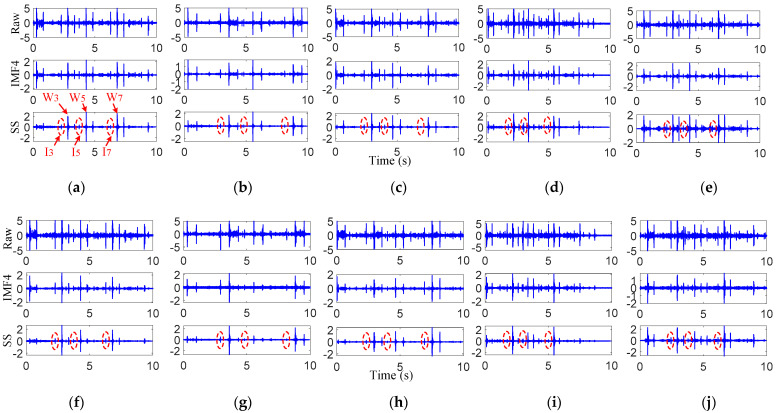
Sensor results of different velocity impact vibrations. Sensor 1: (**a**) 16 km/h, (**b**) 12 km/h, (**c**) 14 km/h, (**d**) 18 km/h, (**e**) 20 km/h; Sensor 2: (**f**) 16 km/h, (**g**) 12 km/h, (**h**) 14 km/h, (**i**) 18 km/h, (**j**) 20 km/h.

Figure 8a shows the raw vibrations, the IMF4 component and further results processed through the SS of the preseted 16 km/h experiment of sensor 1. Compared with the VMD results, three impulses with small amplitudes can be easily identified from the VMD-SS method, which are marked with red dotted lines. To analyze the three small impulses, they were defined as *I*_3_, *I*_5_ and *I_7_*, respectively. Then, the distances between *I*_3_-*W*_3_, *I*_5_-*W*_5_ and *I*_7_-*W*_7_ were calculated with the corrected velocity of 17.1 km/h. The calculated results are shown in the fifth row of Table 4. It can be observed that the distance between the small impulses and the joint was basically consistent with the 2.5 m in Figure 4b. Therefore, we assume that the small impulses were caused by the damage-induced impact vibrations.

To test the repeatability of the results, for the preseted 16 km/h experiment data from sensor 2, and the preseted 12 km/h, 14 km/h, 18 km/h and 20 km/h experiment data from sensor 1–2, the VMD-SS was taken to try to extract impact vibrations. The processing results are shown in Figure 8b–j and Table 4. It should be noted that the corrected velocities of 12 km/h, 14 km/h, 18 km/h and 20 km/h were 13.2 km/h, 14.9 km/h, 18.9 km/h and 21.3 km/h, respectively. As shown in this table, three small impulses can be easily observed at the left sides of the joint, and the distances from the joint were also about 2.5 m, which was the location of the target damage. Therefore, the proposed method can realize damage-induced impact vibration extractions. However, it cannot be ignored that the proposed method does not realize the impact vibration extraction from *W*_4_, *W*_6_ and *W*_8_. One possible reason for these phenomena is that the distance between each two adjacent wheelsets in a bogie is close to 2.5 m. This leads to the damage-induced impact vibrations with small amplitudes being masked by the joint induced impact vibration with large amplitudes from *W*_3_, *W*_5_ and *W*_7_. The other possible reason for these phenomena is the wheel–rail contact states. Under different velocities of the track inspection vehicle, it is impossible to ensure that all wheels have good contact states at the damage and to generate corresponding impact vibrations.

## 4. Influences of Damage Dimensions and Velocities

**Figure 9:**
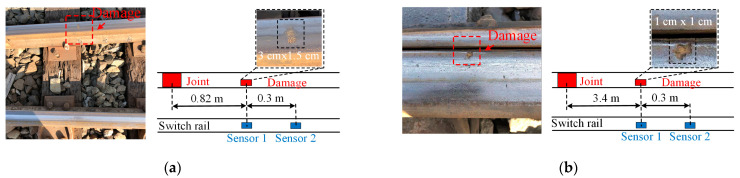
The other damage and location parameters: (**a**) damage 1 and location; (**b**) damage 2 and location.

Considering that in actual scenarios, both damage dimensions and vehicle velocities are varying, it is necessary to analyze the impact vibration features for different damage dimensions under different velocities. In this section, two other head damages are selected to conduct the above analysis, whose locations and dimensions are illustrated in Figure 9a,b. In Figure 9, the damage 1 was 0.82 m away from a left joint, whose dimensions were about 3 cm × 1.5 cm; the damage 2 was 3.4 m away from the left joint, whose dimensions were about 1 cm × 1 cm. The vehicle still passed the damage and nearby joint from left to right with preseted velocities of 12 km/h, 14 km/h, 16 km/h, 18 km/h and 20 km/h, respectively. The sampling frequency was still set at 10 kHz and the sampling time was 10 s.

**Figure 10:**
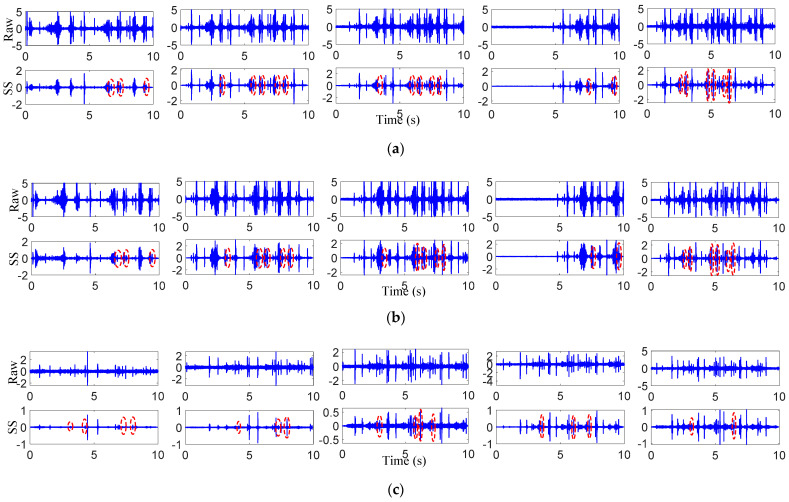

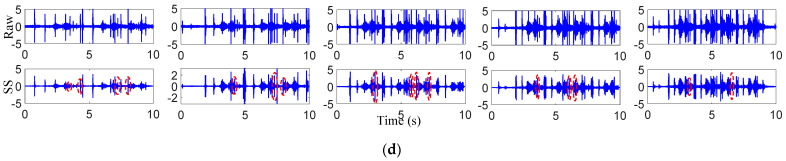
Extracted impact vibrations: (**a**) results of sensor 1 from damage 1; (**b**) results of sensor 2 from damage 1; (**c**) results of sensor 1 from damage 2; (**d**) results of sensor 2 from damage 2.

**Figure 11:**
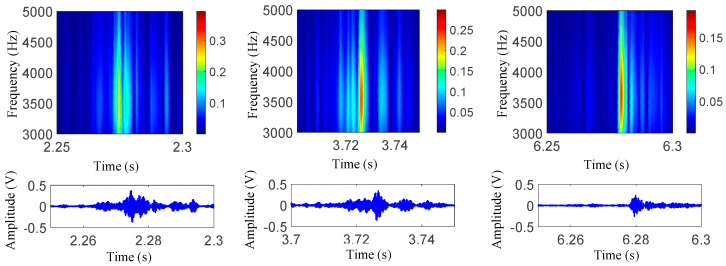
Wavelet analysis examples for impact vibration of sensor 1 under a 12 km/h velocity.

After collecting the experiment signals, the actual velocities were firstly corrected. Specifically, for damage 1 experiments, the corrected velocities were 12.5 km/h, 13.4 km/h, 15.9 km/h, 16.3 km/h and 20.2 km/h, respectively; for damage 2 experiments, the corrected velocities were 11.5 km/h, 13.7 km/h, 15.9 km/h, 18.9 km/h and 21.2 km/h, respectively. Then, the same signal processing method was applied to the data; the corresponding results are shown in Figure 10a–d. As shown in these figures, for damage 1–2, the proposed method can still extract damage-induced small impulses. To further analyze the impact of damage dimensions and velocities on impact vibrations, the frequency and peak-to-peak value features were extracted from the three monitored damage experiments. Among them, the frequency features were extracted through wavelet time–frequency analysis. Figure 11 provides an example of extracted frequency features for damage 1 under the preseted 12 km/h experiment with sensor 1 data. The peak-to-peak features were extracted with the time-domain feature extraction method.

**Figure 12:**
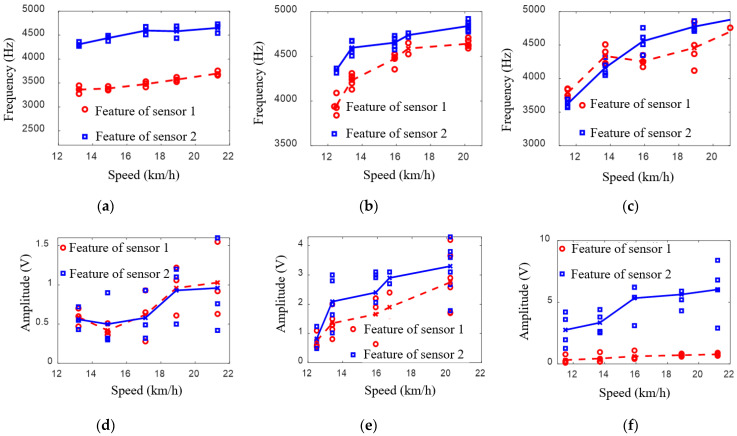
Frequency and amplitude features from different damage: (**a**,**d**) damage 2.5 cm × 2 cm; (**b**,**e**), damage 3 cm × 1.5 cm; (**c**,**f**) damage 1 cm × 1 cm.

Using the above methods, the extracted frequencies and peak-to-peak values are shown in Figure 12a–f. As shown in these figures, for each experiment, the proposed method can identify several damage-induced results. For the convenience of comparison, the mean values of the frequencies and peak-to-peak values are calculated and fitted, in which the sensor 1 results are characterized with red dotted curves, and the sensor 2 results are characterized with blue solid curves.

It can be observed from the frequency curves that the impact vibration frequencies were mainly concentrated in the band of 3500–5000 Hz, and the frequency increased with the vehicle velocity increase. However, it should not be ignored that the monotonic increasing trend of the frequency distribution in Figure 12c was more unstable than those in Figure 12a,b. The possible reason for these phenomena is that the damage dimension of Figure 12c was the smallest of the three damages, meaning the corresponding impact vibration amplitudes may have been the weakest. Therefore, the extracted features were most vulnerable to the complex wheel–rail relationships in switch rail areas and noise. For the peak-to-peak value features, it can be observed from the curves that the values also increased with the vehicle velocity increase. However, by comparing the identified damage features for different dimensions, it can be found that there was no obvious linear relationship between the damage dimensions and peak-to-peak values. The distribution range of the values also had no significant regularity. Specifically, the peak-to-peak values of 2.5 cm × 2 cm damage were distributed at about 0.5–1.5 V; the peak-to-peak values of 3 cm × 1.5 cm damage were distributed at about 0.5–4.5 V; and the peak-to-peak values of 1 cm × 1 cm damage were distributed at about 0.5–8 V. The possible reasons for these phenomena are: (1) the varying wheel–rail contact states in different switch rail areas and the coupling states between sensors and rail bases were different, which led to the change in the impact vibration amplitudes; (2) there was still noise in the processed signals in Figure 8 and Figure 10, which would affect the accuracy of the feature extractions.

## 5. Conclusions

In this paper, a damage-induced switch rail impact vibration extraction method is proposed based on VMD-SS. It is aimed at the challenge that the nonstationary vibration signals in switch rail areas make it difficult to characterize damage and structural states. In a practical switch rail line, with the help of a track inspection car, the proposed method was verified with different damage dimensions under different velocities.

The result showed that under the scenario of less than 20 km/h, (1) the proposed signal processing method based on VMD-SS can realize impact vibration extractions; (2) the impact vibration frequencies are mainly concentrated in the band of 3500–5000 Hz, and the frequency increases with the vehicle velocity increase; and (3) the impact vibration peak-to-peak values also increase with the vehicle velocity increase. However, there are no obvious linear relationships between the damage dimensions and peak-to-peak values. This may be due to the wheel–rail contact states, the sensor coupling and the low signal-to-noise ratio.

The next work will consider the modeling of adding wheel–rail rolling contacts, involve experiments with higher velocities and further verify the proposed method. We will also improve the signal-to-noise ratio of the extracted impact vibrations.

## Figures and Tables

**Table 1:** The material parameters of simulation model.

Young’s Modulus/GPA	Poisson’s Ratio	Density/kg/m^3^
206	0.3	7850

**Table 2:** The calculation results of wave peaks spacing after velocity correction.

	$d_{W_{23}}/m$	$d_{W_{34}}/m$	$d_{W_{45}}/m$	$d_{W_{56}}/m$	$d_{W_{67}}/m$	$d_{W_{78}}/m$
Calculated value	9.44	2.58	4.44	2.62	9.39	2.57
Actual value	9.4	2.6	4.46	2.6	9.4	2.6
Error	0.43%	0.77%	0.45%	0.77%	0.1%	1.1%

**Table 3:** The central frequencies of different IMF orders.

IMF Component Number	Central Frequency of Modal Component/Hz				
	IMF1	IMF2	IMF3	IMF4	IMF5
2	33	1229			
3	33	1229	2900		
4	33	1229	2900	3655	
5	33	1229	2611	2900	3655

**Table 4:** The calculated results of impact vibrations.

Preseted Velocity /km/h	Wave Crest	Measuring Point 1			Measuring Point 2		
		I3	I5	I7	I3	I5	I7
12	Calculated value	2.49 m	2.62 m	2.47 m	2.55 m	2.65 m	2.48 m
	Error	0.4%	4.8%	1.1%	2%	6%	0.8%
14	Calculated value	2.53 m	2.68 m	2.5 m	2.56	2.68	2.5
	Error	1.2%	7.2%	0%	2.4%	7.2%	0%
16	Calculated value	2.55	2.69	2.52	2.49	2.64	2.49
	Error	2%	7.6%	0.8%	0.4%	5.6%	0.4%
18	Calculated value	2.56	2.71	2.57	2.55	2.7	2.56
	Error	2.4%	8.4%	2.8%	2%	8%	2.4%
20	Calculated value	2.58	2.7	2.56	2.55	2.65	2.53
	Error	3.2%	8%	2.4%	2%	6%	1.2%

## Data Availability

Not applicable.
